# Radioactive iodine in low- to intermediate-risk papillary thyroid cancer

**DOI:** 10.3389/fendo.2022.960682

**Published:** 2022-08-11

**Authors:** Hengqiang Zhao, Yiping Gong

**Affiliations:** Department of Breast and Thyroid Surgery, Renmin Hospital of Wuhan University, Wuhan, China

**Keywords:** papillary thyroid carcinoma, total thyroidectomy, radioactive iodine, prognosis, propensity score matching

## Abstract

It remains controversial whether papillary thyroid cancer (PTC) patients with low- to intermediate-risk disease should receive radioactive iodine (RAI) after total thyroidectomy (TT). We aim to identify those who might benefit from RAI treatment in PTC patients with cervical nodal metastasis after TT. Patients were divided into TT and TT+RAI groups from the Surveillance, Epidemiology, and End Results (SEER) database (2004–2018). Overall survival (OS) and cancer-specific survival (CSS) were compared, and propensity score matching (PSM) was performed between groups. A total of 15,179 patients were enrolled, including 3,387 (22.3%) who underwent TT and 11,792 (77.7%) who received TT+RAI. The following characteristics were more likely to present in the TT+RAI group: multifocality, capsular extension, T3, N1b, and more metastatic cervical lymph nodes. RAI was associated with better OS in low- to intermediate-risk PTC patients in the multivariate Cox regression model. The subgroup analysis showed that RAI predicted better OS in patients ≥55 years, American Joint Committee on Cancer (AJCC) stage II, and capsular extension with a hazard ratio (HR) (95% CI) of 0.57 (0.45–0.72), 0.57 (0.45–0.72), and 0.68 (0.51–0.91), respectively. However, RAI failed to improve the prognoses of patients with age <55 years, AJCC stage I, PTC ≤1 cm, and capsular invasion. In the PSM cohort with 3,385 paired patients, TT+RAI treatment predicted better OS compared with TT alone. In addition, TT+RAI predicted better OS in patients with metastatic cervical lymph nodes ≥2, multifocality, extracapsular extension, and American Thyroid Association (ATA) intermediate risk. In conclusion, RAI was associated with better OS in low- to intermediate-risk PTC patients with age ≥55 years, multifocality, extrathyroidal extension, and ATA intermediate risk. However, the survival benefit from RAI may be limited in patients with AJCC stage I, PTC ≤1 cm, unifocality, capsular invasion, and ATA low-risk diseases; these patients even showed pathological cervical lymph node metastasis.

## Introduction

The incidence of papillary thyroid carcinoma (PTC) has increased substantially in recent decades (1). PTC is the most frequent subtype of thyroid cancer, accounting for more than 90% of all thyroid cancers. The standard treatment toward PTC includes thyroid surgery, thyrotropin inhibition, and radioactive iodine (RAI) (2). Thyroidectomy and thyroid hormone therapy are routinely performed on PTC patients. However, the administration of RAI is mainly based on postoperative risk stratification.

RAI is routinely recommended for high-risk PTC patients after total thyroidectomy (TT), while RAI is not recommended for papillary thyroid microcarcinoma (PTMC) patients in the absence of other adverse features according to the American Thyroid Association (ATA) guidelines (3). More specifically, patients with gross extrathyroidal extension and distant metastases were recommended for RAI, while patients with PTMC in the absence of cervical lymph node and distant metastases were not recommended for RAI therapy (3).

However, PTC patients with low to intermediate risk are not routinely recommended for RAI for conflicting observational evidence, and thus more research is needed to uncover the therapeutic efficacy in various subgroups of patients in the ATA low- to intermediate-risk category (3, 4). In the present study, we aimed to investigate the effect of RAI on the prognosis of PTC patients with cervical lymph node metastasis after TT. We collected the data from the largest public database and balanced the variables between groups using propensity score matching (PSM) method. The present study provided new evidence for better treatment of PTC.

## Patients and methods

### Ethics statement

Patients were obtained from the Surveillance, Epidemiology, and End Results (SEER) database from 2004 to 2018 (SEERStat user name: 21208-Nov2020). The SEER database collected patients with deidentified information; thus, this study was granted exempt status by our institutional review board.

### Study population

The SEER*Stat Database named “Incidence-SEER Research Plus Data, 18 Registries, Nov 2020 Sub (2000–2018)” was selected for the following research. The exclusion criteria were as follows: patients with multiple (≥2) primary tumors, age <18 years, non-positive histology, chemotherapy, and distant metastasis. All patients underwent TT and cervical lymph node dissection. Patients with T4 were excluded. In the present study, tumor extrathyroidal extension represents those with minimal extrathyroidal extension including strap muscles and pericapsular soft tissue. Only patients with cervical lymph node metastasis were included, and cervical lymph node metastases were categorized into N1a (central compartment lymph node metastases), N1b (lateral neck lymph node metastases), and N1NOS. Patients with unknown or indefinite information on the included variables such as tumor number, capsular extension, tumor stage, T/N stage, RAI therapy, cause-specific death, lymph node examined, and positive lymph nodes were excluded. Patients with International Classification of Diseases for Oncology (third edition) codes of 8050, 8260, 8340, 8341, 8342, 8343, 8344, and 8350 were enrolled. Tumor stage was based on the American Joint Committee on Cancer (AJCC) eighth edition. Patients were divided into three races: White, Black, and Other (Asian or Pacific Islander and American Indian/Alaska Native).

### Statistical analysis

The chi-square test, Mann–Whitney *U* test, and Student’s t-test were used to analyze the category variables, data with skewed distribution (age and year of diagnosis), and the number of lymph nodes between groups, respectively. Kaplan–Meier curves of cancer-specific survival (CSS) and overall survival (OS) were analyzed using log-rank test. Risk factors for CSS and OS were estimated by Cox proportional hazards model with hazard ratio (HR) and a 95% confidence interval (CI).

PSM analysis was performed between groups to balance the statistical differences of the clinicopathologic features such as year of diagnosis, multifocality, capsular extension, T/N stage, and positive regional lymph nodes between TT and TT+RAI groups. PSM of 1:1 matching with a caliper of 0.1 was selected using R software (ver. 3.3.3) as we previously described (5). Two-sided *P* < 0.05 was considered statistically significant.

## Results

### Patient characteristics

**Figure 1:**
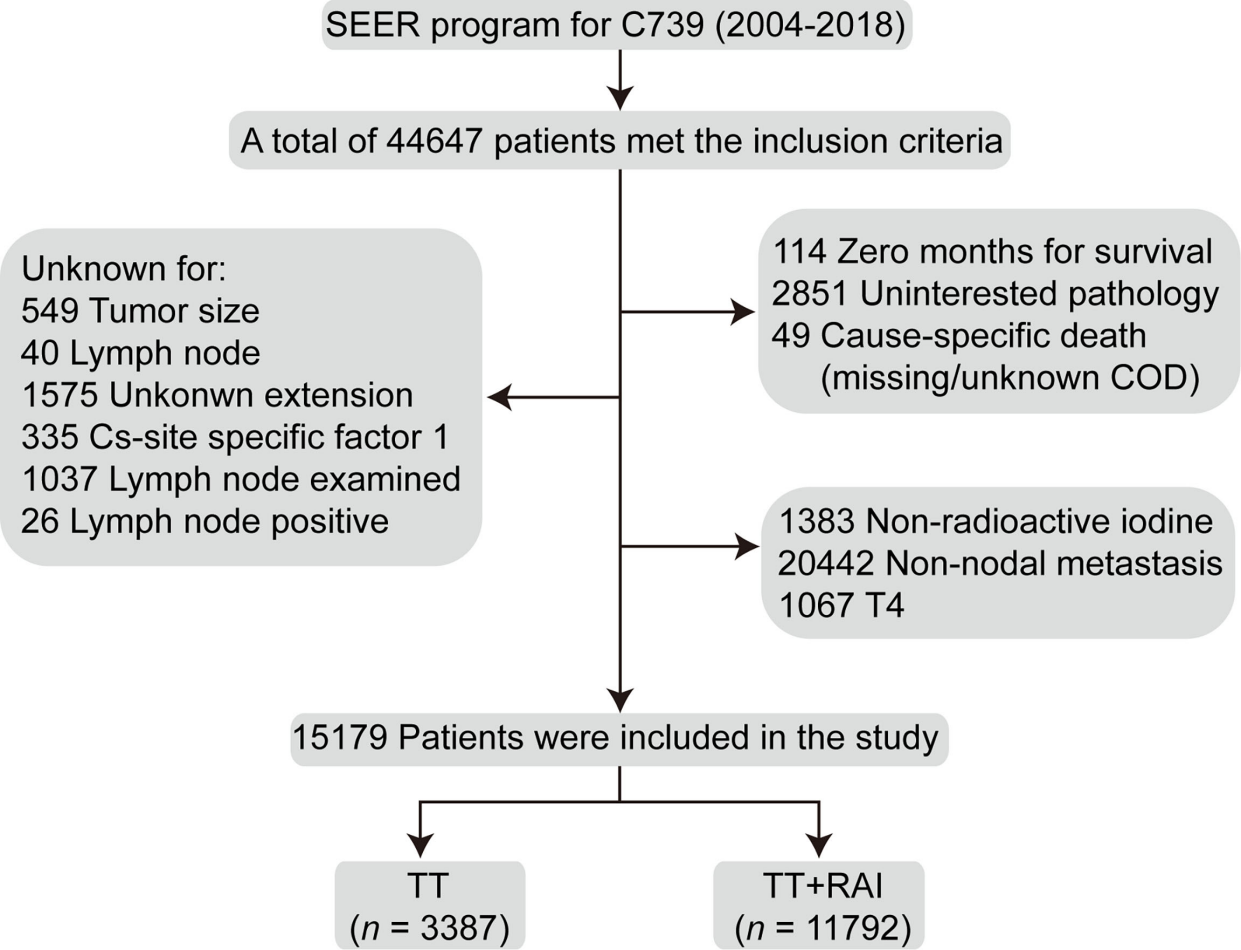
Flowchart of the selection process of the patients who underwent TT and additional RAI treatment from the Surveillance, Epidemiology, and End Results database. TT, total thyroidectomy; RAI, radioactive iodine.

The flowchart of the selection process was shown in **Figure 1**. Finally, a total of 15,179 patients were enrolled, including 3,387 (22.3%) with TT alone and 11,792 (77.7%) receiving additional RAI therapy (**Table 1**). The following characteristics of patients were more likely to present in the TT+RAI group compared with the TT group: multifocality, extracapsular extension, T3, N1b, more cervical lymph node examined, and more metastatic cervical lymph nodes (**Table 1**).

**Table 1:** Characteristics of the study population (*n* = 15,179).

Variables	TT (*n* = 3,387	TT+RAI (*n* = 11,792)	*P*-value
Age	41 (32–52)	41 (32–52)	0.426
<55 years	2,698 (79.7)	9,475 (80.4)	0.372
≥55 years	689 (20.3)	2,317 (19.6)	
Year of diagnosis	2011 (2008–2013)	2011 (2008–2013)	0.036
Sex			
Men	924 (27.3)	3,400 (28.8)	0.078
Women	2,463 (72.7)	8,392 (71.2)	
Race			
White	2,832 (83.6)	9,795 (83.1)	0.410
Black	104 (3.1)	334 (2.8)	
Other	451 (13.3)	1,663 (14.1)	
Multifocality			
No	1,541 (45.5)	5,017 (42.5)	0.002
Yes	1,846 (54.5)	6,775 (57.5)	
Capsular extension			
Intrathyroidal tumor	2,161 (63.8)	6,670 (56.6)	<0.001
Capsular invasion	272 (8.0)	905 (7.7)	
Extracapsular extension	954 (28.2)	4,217 (35.8)	
AJCC stage eighth			
I	2,698 (79.7)	9,475 (80.4)	0.372
II	689 (20.3)	2,317 (19.6)	
T stage			
T1	1,589 (46.9)	4,836 (41.0)	<0.001
T2	654 (19.3)	2,187 (18.5)	
T3	1,144 (33.8)	4,769 (40.4)	
N stage			
N1a	2,055 (60.7)	6,821 (57.8)	<0.001
N1b	937 (27.7)	3,886 (33.0)	
N1NOS	395 (11.7)	1,085 (9.2)	
CLN examined	13.5 ± 16.9	14.9 ± 17.9	<0.001
CLN positive	4.7 ± 6.0	5.4 ± 6.2	<0.001
Number of deaths			
CSS	40	126	–
OS	149	345	–

Age and year of diagnosis were expressed as median with interquartile range; other variables were expressed as n (%).

TT, total thyroidectomy; RAI, radioactive iodine; CLN, cervical lymph node; CSS, cancer-specific survival; OS, overall survival.

### Predictors for cancer-specific survival and overall survival of patients with papillary thyroid microcarcinoma

After adjustment for patient age, year of diagnosis, sex, race, capsular extension, multifocality, T/N stage, cervical lymph node examined, and positive cervical lymph nodes in the multivariate Cox regression model, RAI treatment was associated with better OS with an HR (95% CI) of 0.66 (0.54–0.80), while RAI failed to improve CSS with an HR (95% CI) of 0.89 (0.62–1.27) compared with TT treatment alone (**Table 2**).

**Table 2:** Multivariate Cox regression analysis for cancer-specific survival and overall survival of PTC patients.

Category	Cancer-specific survival		Overall survival	
	HR (95% CI)	*P*-value	HR (95% CI)	*P-*value
Age	1.11 (1.10–1.12)	<0.001	1.10 (1.09–1.10)	<0.001
Year of diagnosis	0.94 (0.89–0.99)	0.030	0.96 (0.93–0.99)	0.012
Sex				
Men	Ref		Ref	
Women	0.84 (0.61–1.16)	0.286	0.58 (0.48–0.69)	<0.001
Race				
White	Ref		Ref	
Black	2.07 (0.96–4.47)	0.063	1.83 (1.18–2.85)	0.007
Other	0.99 (0.64–1.53)	0.950	0.78 (0.58–1.04)	0.090
Multifocality				
No	Ref		Ref	
Yes	0.78 (0.57–1.06)	0.108	0.97 (0.81–1.16)	0.748
Capsular extension				
Intrathyroidal tumor	Ref		Ref	
Capsular invasion	1.22 (0.62–2.41)	0.566	1.03 (0.71–1.49)	0.870
Extracapsular extension	0.66 (0.38–1.14)	0.132	0.85 (0.57–1.27)	0.431
T stage				
T1	Ref		Ref	
T2	2.60 (1.45–4.65)	0.001	1.05 (0.79–1.39)	0.757
T3	6.25 (3.26–11.97)	<0.001	1.67 (1.12–2.51)	0.013
N stage				
N1a	Ref		Ref	
N1b	1.62 (1.11–2.37)	0.013	1.10 (0.89–1.37)	0.371
N1NOS	1.86 (1.15–3.00)	0.011	1.16 (0.86–1.55)	0.332
RAI				
No	Ref		Ref	
Yes	0.89 (0.62–1.27)	0.524	0.66 (0.54–0.80)	<0.001
CLN examined	1.00 (0.99–1.01)	0.752	1.00 (0.99–1.01)	0.907
CLN positive	1.05 (1.02–1.08)	<0.001	1.04 (1.02–1.06)	<0.001

PTC, papillary thyroid cancer; HR (95% CI), hazard ratio (95% confidence interval); RAI, radioactive iodine; CLN, cervical lymph node.

### Subgroup analysis

The subgroup analyses stratified by the clinicopathologic characteristics were performed to assess the robustness of the results. TT+RAI treatment was associated with better OS in most subgroups (**Table 3**). However, RAI use was not associated with better OS in patients with age <55 years, black patients, AJCC stage I, PTMC, and capsular invasion with HRs (95% CI) of 0.88 (0.62–1.26), 0.95 (0.30–2.97), 0.88 (0.62–1.26), 0.79 (0.49–1.25), and 0.97 (0.42–2.24), respectively, compared with TT alone. In addition, RAI was not associated with better CSS in the subgroup analyses. We further found that PTMC patients failed to gain better CSS and OS with the addition of RAI treatment. Patients were divided into low-risk and intermediate-risk subgroups according to ATA risk stratification. Compared with TT alone, TT+RAI treatment was associated with better OS in ATA low-risk and intermediate-risk subgroups with HRs (95% CI) of 0.71 (0.51–0.97) and 0.63 (0.49–0.80), respectively (**Table 3**).

**Table 3:** Subgroup analysis for cancer-specific survival and overall survival of TT with RAI vs. TT alone by multivariate Cox regression analysis.

Category	Cancer-specific survival		Overall survival	
	HR (95% CI)	*P*-value	HR (95% CI)	*P*-value
Age				
<55 years	1.37 (0.57–3.29)	0.478	0.88 (0.62–1.26)	0.493
≥55 years	0.72 (0.48–1.07)	0.102	0.57 (0.45–0.72)	<0.001
Sex				
Men	0.78 (0.46–1.32)	0.354	0.60 (0.46–0.79)	<0.001
Women	0.98 (0.60–1.62)	0.946	0.71 (0.54–0.95)	0.018
Race				
White	0.87 (0.58–1.29)	0.481	0.64 (0.52–0.78)	<0.001
Black	0.74 (0.07–7.74)	0.802	0.95 (0.30–2.97)	0.927
Multifocality				
No	0.95 (0.56–1.64)	0.864	0.67 (0.49–0.91)	0.009
Yes	0.85 (0.52–1.38)	0.508	0.65 (0.50–0.84)	0.001
Capsular extension				
Intrathyroidal tumor	0.81 (0.43–1.53)	0.523	0.61 (0.46–0.81)	0.001
Capsular invasion	3.21 (0.37–27.85)	0.290	0.97 (0.42–2.24)	0.949
Extracapsular extension	0.81 (0.51–1.27)	0.353	0.68 (0.51–0.91)	0.009
AJCC stage eighth				
I	1.37 (0.57–3.29)	0.478	0.88 (0.62–1.26)	0.493
II	0.72 (0.48–1.07)	0.102	0.57 (0.45–0.72)	<0.001
T stage				
PTMC	1.44 (0.41–5.10)	0.568	0.79 (0.49–1.25)	0.307
T1	1.07 (0.39–2.92)	0.890	0.71 (0.51–0.99)	0.045
T2	0.89 (0.32–2.48)	0.829	0.53 (0.32–0.88)	0.015
T3	0.82 (0.54-1.25)	0.351	0.67 (0.51–0.88)	0.004
N stage				
N1a	1.47 (0.74–2.93)	0.270	0.72 (0.54–0.95)	0.021
N1b	0.64 (0.39–1.04)	0.072	0.59 (0.43–0.81)	0.001
ATA risk stratification				
ATA low risk	1.83 (0.71–4.78)	0.212	0.71 (0.51–0.97)	0.032
ATA intermediate risk	0.75 (0.51–1.11)	0.148	0.63 (0.49–0.80)	<0.001

HRs were adjusted for age, year of diagnosis, sex, race, multifocality, capsular extension, T/N stage, RAI, and number of metastatic lymph nodes.

RAI, radioactive iodine; TT, total thyroidectomy; PTC, papillary thyroid cancer; HR (95% CI), hazard ratio (95% confidence interval); PTMC, papillary thyroid microcarcinoma; ATA, American Thyroid Association.

### Kaplan–Meier curves before and after propensity score matching

**Figure 2:**
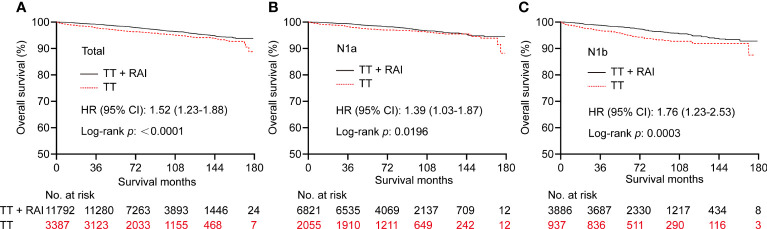
Kaplan–Meier curves of OS in PTC patients who underwent TT vs. TT+RAI before PSM in total patients **(A)**, N1a patients **(B)**, and N1b patients **(C)**. OS, overall survival; PTC, papillary thyroid carcinoma; TT, total thyroidectomy; RAI, radioactive iodine; PSM, propensity score matching.

Kaplan–Meier curves showed that RAI therapy was associated with better OS with an HR (95% CI) of 1.52 (1.23–1.88) before PSM (**Figure 2A**). We further divided patients into N1a and N1b subsets. Similarly, RAI was associated with better OS in patients with N1a with an HR (95% CI) of 1.39 (1.03-1.87) (**Figure 2B**), and in patients with N1b with an HR (95% CI) of 1.76 (1.23–2.53) (**Figure 2C**).

**Figure 3:**
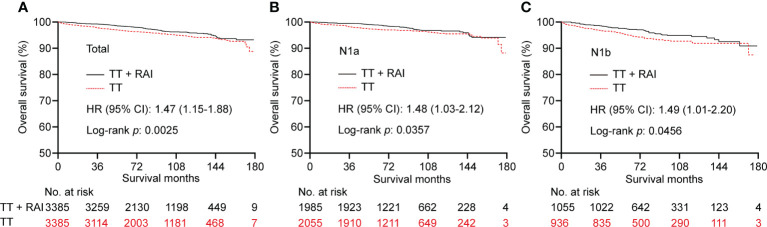
Kaplan–Meier curves of OS in PTC patients who underwent TT vs. TT+RAI after PSM in total patients **(A)**, N1a patients **(B)**, and N1b patients **(C)**. OS, overall survival; PTC, papillary thyroid carcinoma; TT, total thyroidectomy; RAI, radioactive iodine; PSM, propensity score matching.

To increase the between-group comparability, we performed PSM analysis to balance the clinicopathologic characteristics between TT and TT+RAI groups. The PSM process yielded 3,385 paired patients. After PSM, the differences of baseline characteristics between the two groups were obviously reduced (**Supplementary Table S1**). After PSM, RAI was associated with better OS with an HR (95% CI) of 1.47 (1.15–1.88) (**Figure 3A**). Consistently, RAI was associated with better OS in N1a patients with an HR (95% CI) of 1.48 (1.03–2.12) and in N1b patients with an HR (95% CI) of 1.49 (1.01–2.20) (**Figures 3B, C**).

**Figure 4:**
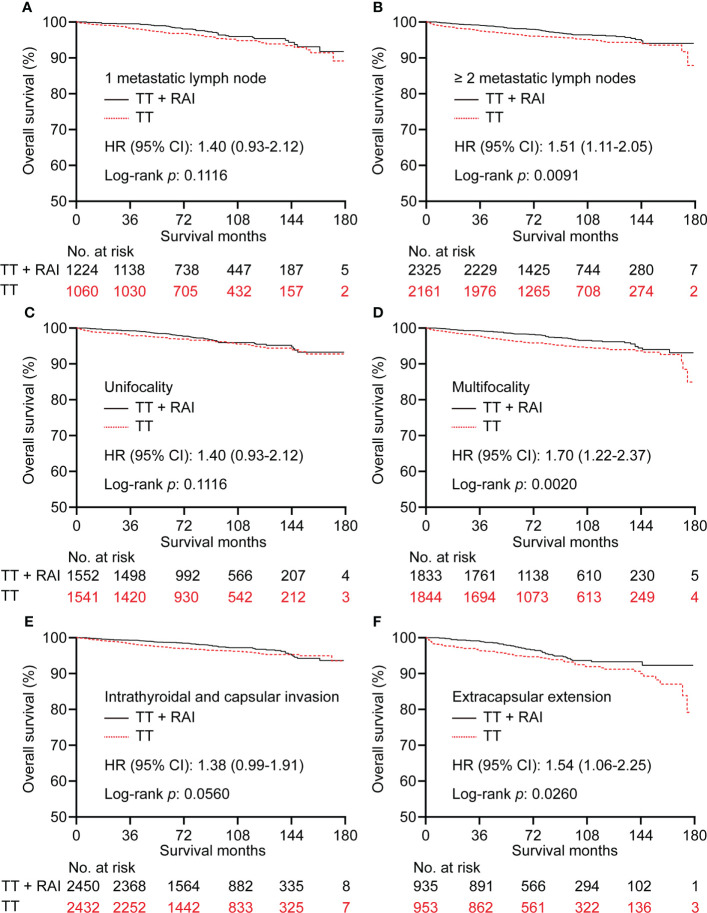
Kaplan–Meier curves of OS in PTC patients who underwent TT vs. TT+RAI after PSM stratified by the number of metastatic lymph nodes **(A, B)**, tumor number **(C, D)**, and tumor extension **(E, F)**. OS, overall survival; PTC, papillary thyroid carcinoma; TT, total thyroidectomy; RAI, radioactive iodine; PSM, propensity score matching.

**Figure 5:**
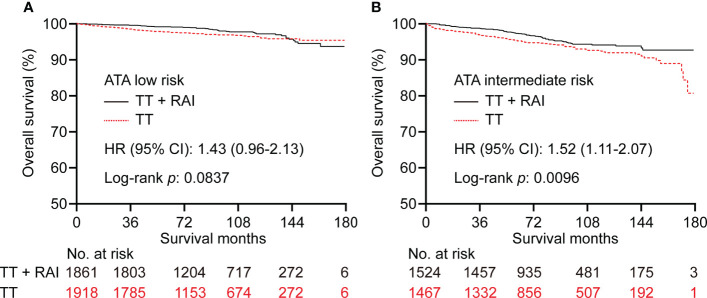
Kaplan–Meier curves of OS in PTC patients underwent TT vs. TT+RAI after PSM in low-risk **(A)** and intermediate-risk **(B)** patients stratified by the ATA risk stratification. OS, overall survival; PTC, papillary thyroid carcinoma; TT, total thyroidectomy; RAI, radioactive iodine; PSM, propensity score matching; ATA, American Thyroid Association.

In the PSM cohort, we further stratified patients into one metastatic lymph node vs. ≥2 metastatic lymph nodes, unifocality vs. multifocality, extracapsular extension vs. intrathyroidal and capsular invasion tumor. There were no significant statistical differences in OS between TT+RAI and TT groups in patients with one metastatic lymph node, unifocality, and intrathyroidal and capsular invasion tumor (**Figures 4A, C, E**). Compared with TT+RAI, TT alone was associated with poorer OS in patients with ≥2 metastatic lymph nodes, multifocality, and extracapsular extension with HRs (95% CI) of 1.51 (1.11–2.05), 1.70 (1.22–2.37), and 1.54 (1.06–2.25), respectively (**Figures 4B, D, F**). We further divided patients into ATA low-risk vs. ATA intermediate-risk subgroups. In the ATA low-risk patients, there was no significant difference in OS between patients with TT and TT+RAI with an HR (95% CI) of 1.43 (0.96–2.13) (**Figure 5A**). In the ATA intermediate-risk subgroup, TT was associated with poorer OS compared with TT+RAI treatment with an HR (95% CI) of 1.52 (1.11–2.07) (**Figure 5B**).

## Discussion

In this study, we examined the effect of RAI treatment on the prognoses of PTC patients who underwent TT and cervical lymph node dissection. All of the enrolled patients showed cervical lymph node metastasis and were not diseases of T4 and/or distant metastasis. We found that RAI+TT treatment was associated with better OS compared with TT alone before and after PSM in the low- to intermediate-risk PTC patients. The subgroup analyses showed that RAI was associated with better OS in patients with age ≥55 years, AJCC stage II, and extracapsular extension. However, RAI failed to improve prognoses in patients with age <55 years, AJCC stage I, and capsular invasion compared with the TT group. In the PSM cohort, Kaplan–Meier curves showed that RAI treatment was associated with better OS in patients with ≥2 metastatic lymph nodes, multifocality, extracapsular extension, and ATA intermediate risk.

The difficulty in performing RAI is to identify patients who are most likely to benefit from RAI therapy while avoiding unnecessary exposure to ionizing radiation in the majority of low- to intermediate-risk thyroid cancer patients (6). Except for the strong recommendation for RAI treatment in patients with gross tumor extension and distant metastases and non-recommendation for RAI in PTMC patients without risk clinicopathologic features, RAI use remains controversial in most cases (7). Consistent with the ATA recommendation, our study found that PTMC patients did not benefit from RAI administration, while patients with extrathyroidal extension had better OS with the addition of RAI. A previous study showed that differentiated thyroid cancer ≤2 cm without risk factors such as multifocality, extrathyroidal extension, lymph node, and/or distant metastasis may exempt from RAI and receive less extensive surgery (8).

In our study, about 80% of patients were AJCC stage I (eighth edition), and these patients failed to benefit from RAI administration. Of note, these patients were those <55 years for the enrollment of patients with nodal metastasis in our study. On the contrary, patients with older age (≥55 years) benefited from RAI therapy with the improvement of OS, which may reflect the survival benefit from RAI in patients with AJCC stage II. One important finding in our study was that both patients with N1a and N1b benefited from RAI therapy. In clinical practice, the proportion of N1b subtype was about 13.5% in PTC patients (9), and about 50% of PTC patients present with pathological N1a disease (10). Increasing metastatic lymph nodes were associated with compromised survival in PTC patients (11). Therefore, we can expect that a change point of the number of metastatic lymph nodes in the central compartment may exist, and patients with more metastatic lymph nodes were more likely to benefit from RAI treatment. In the PSM cohort, the mean and median number of metastatic lymph nodes were 5.2 and 3.0, respectively. We found that PTC patients with ≥2 metastatic lymph nodes can benefit from RAI use, while patients with only one metastatic lymph node failed to benefit from RAI treatment. This should be validated in the following studies. A recent study by Sun et al. (12) found that RAI therapy predicted better OS in both N1a and N1b subtypes in the multivariate Cox regression model, which was consistent with our results. However, they did not examine the robustness of results stratified by N1a and N1b subsets in a PSM cohort (12). Wang et al. (13) found that there was no difference in the 5-year central compartment nodal recurrence-free survival between PTC patients treated with RAI and those without RAI treatment. However, the 104 patients and the median follow-up of 53 months may be limited in the evaluation of the recurrence risk of PTC (13). A recent study showed that the recurrence-free survival curve of the intermediate-risk PTC patients who received postoperative RAI therapy (n = 208) did not differ significantly from that of the patients who did not receive it (n = 141), especially for patients with negative extranodal spread and low number (<5) of metastatic lymph nodes (14). However, they did not focus on OS of PTC patients.

New evidence showed that RAI utilization declined from 61.0% in 2004 to 43.9% in 2016, especially in patients with T1a (tumor ≤1 cm in diameter), N0/X, M0 PTC without extrathyroidal extension (34.8% in 2004 to 9.5% in 2015) in the United States (15). Similarly, a recent study showed that the declining use of RAI represented the most pronounced change in the management of PTCs <4 cm (44%–18% during the period 2006–2018), including PTMC (26%–6% during the period 2007–2018). For pediatric patients (<20 years), use of RAI peaked in 2009 (59%), then decreased markedly to 11% (2018) (16). A recent survey showed that more than half of the patients (55.8%) feel that they did not have an RAI choice, while the majority of patients (75.9%) received RAI, suggesting a need for more shared decision-making to reduce overtreatment (17). Nearly 25% of low-risk PTC patients (defined T1 without metastasis) received RAI. Predictors of overtreatment with RAI included age <45 years, age 45–64 years, male sex, Hispanic and Asian race, and extensive lymphadenectomy (18). Efforts to reduce the overuse of RAI in low-risk thyroid cancer should include interventions targeted toward physicians (19). It seems apparent that RAI administration changes with the implementation and dissemination of evidence-based guidelines toward PTC with RAI use, and prudent use of RAI should be considered in low-risk patients (20).

Our study has several advantages compared with previous studies. Firstly, we used the largest database with more pathological variables included such as tumor multifocality, extrathyroidal extension, and metastatic lymph nodes, and variables with unknown information were excluded compared with previous studies with the same topic (12, 21). In addition, we adopted the updated AJCC stage to assess the therapeutic effect of RAI use for the first time. We further validated the limited survival benefit in PTMC patients, which might reduce the potentially unnecessary RAI use in the majority of PTMC patients. We also demonstrated that patients with minimal extrathyroidal extension gained better OS after RAI use. Additionally, we used the PSM analysis to balance the clinicopathologic differences between groups; this procedure improved the comparability and reliability of the results. However, some limitations must be acknowledged. The retrospective design prevented proving causality between RAI use and the better prognosis of PTC patients. However, the population-based nature may reflect the “real-world” management outcomes found outside of the well-controlled trials. In addition, the SEER database cannot evaluate patients’ comorbidities and disease recurrence. Additionally, the dosages of RAI were not recorded in the SEER database. We therefore cannot determine the optimal dosages of RAI that will benefit PTC patients most. Last but not least, TT+RAI treatment was not associated with better CSS compared with TT alone in this study. This may be caused by the excellent prognosis of PTC and limited follow-up period. RAI treatment should be narrowed down to only those cases that will truly benefit from RAI treatment. Nowadays, the usual medical practice is to conduct a genetic search for drug therapy and decide whether to treat the patient according to the results of the companion diagnosis. The amount of sodium/iodide symporter (NIS) expression in tumor tissue and the presence or absence of genetic abnormalities that could alter NIS expression should be used as indicators to determine future indications for RAI treatment (22). Apart from postoperative risk assessment, radioactive iodine imaging, preferences of local disease management, assessment of potential side effect, and patients’ preferences are additional key elements that must be considered when deciding whether an individual patient could benefit from RAI treatment (23).

In conclusion, this PSM-based study suggested that RAI treatment predicted better OS in low- to intermediate-risk PTC patients, especially in those with advanced diseases like AJCC stage II, age ≥55 years, multifocality, extracapsular extension, and ATA intermediate-risk, while RAI may not bring survival benefit in patients with age <55 years, AJCC stage I, capsular invasion, PTMC, and ATA low-risk diseases. A large cohort study with a longer follow-up period is warranted to confirm the present findings.

## Data availability statement

The original contributions presented in the study are included in the article/**Supplementary Material**. Further inquiries can be directed to the corresponding authors.

## Ethics statement

Ethical review and approval was not required for the study on human participants in accordance with the local legislation and institutional requirements. Written informed consent for participation was not required for this study in accordance with the national legislation and the institutional requirements.

## Author contributions

HZ: conception, data acquisition. HZ and YG: data analysis and drafting the article. HZ and YG: revised it critically for important intellectual content. HZ: investigation, project administration, and supervision. Both authors read and approve the final version of the manuscript.

## Funding

This study was supported by Open Foundation of Hubei Key Laboratory of Renmin Hospital of Wuhan University (grant number 2021KFY009).

## Conflict of interest

The authors declare that the research was conducted in the absence of any commercial or financial relationships that could be construed as a potential conflict of interest.

## Publisher’s note

All claims expressed in this article are solely those of the authors and do not necessarily represent those of their affiliated organizations, or those of the publisher, the editors and the reviewers. Any product that may be evaluated in this article, or claim that may be made by its manufacturer, is not guaranteed or endorsed by the publisher.
