# Supplementary material for: Radioactive iodine in low- to intermediate-risk papillary thyroid cancer
Source: Front Endocrinol (Lausanne). 2022 Aug 11;13:960682. doi: 10.3389/fendo.2022.960682 (PMC9402902; doi:10.3389/fendo.2022.960682)
Supplement: Supplementary file 1 [file Table_1.docx]

Supplementary Material

| **Supplementary Table 1** Characteristics of the study population after PSM. | | | |
| --- | --- | --- | --- |
| Variables | TT (*n* = 3385) | TT+RAI (*n* = 3385) | *P*-value |
| Age | 41 (32-52) | 41 (32-52) | 0.644 |
| Year of diagnosis | 2011 (2008-2013) | 2011 (2008-2013) | 0.741 |
| Sex |  |  |  |
| Male | 923 (27.3) | 993 (29.3) | 0.059 |
| Female | 2462 (72.7) | 2392 (70.7) |  |
| Race |  |  |  |
| White | 2830 (83.6) | 2806 (82.9) | 0.569 |
| Black | 104 (3.1) | 99 (2.9) |  |
| Other | 451 (13.3) | 480 (14.2) |  |
| Multifocality |  |  |  |
| No | 1541 (45.5) | 1552 (45.8) | 0.788 |
| Yes | 1844 (54.5) | 1833 (54.2) |  |
| Capsular extension |  |  |  |
| Intrathyroidal tumor | 2160 (63.8) | 2166 (64.0) | 0.803 |
| Capsular invasion | 272 (8.0) | 284 (8.4) |  |
| Capsular extension | 953 (28.2) | 935 (27.6) |  |
| AJCC stage 8th |  |  |  |
| I | 2696 (79.6) | 2725 (80.5) | 0.378 |
| II | 689 (20.4) | 660 (19.5) |  |
| Tumor |  |  |  |
| T1 | 1589 (46.9) | 1588 (46.9) | 0.375 |
| T2 | 653 (19.3) | 694 (20.5) |  |
| T3 | 1143 (33.8) | 1103 (32.6) |  |
| N stage |  |  |  |
| N1a | 2055 (60.7) | 1985 (58.6) | 0.003 |
| N1b | 936 (27.7) | 1055 (31.2) |  |
| N1NOS | 394 (11.6) | 345 (10.2) |  |
| CLN examined | 13.5±16.8 | 13.8±17.2 | 0.315 |
| CLN positive | 4.6±5.8 | 4.8±5.6 | 0.378 |
| TT, total thyroidectomy; RAI, radioactive iodine; PSM, propensity score matching; CLN, cervical lymph node. | | | |
